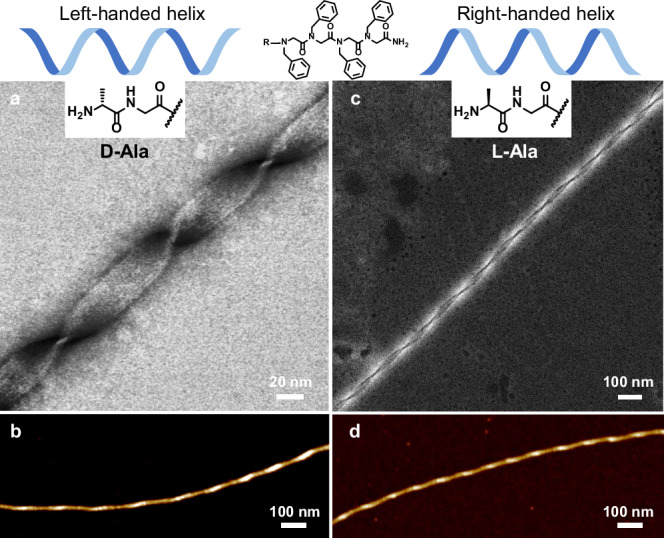# Publisher Correction: Assembly of short amphiphilic peptoids into nanohelices with controllable supramolecular chirality

**DOI:** 10.1038/s41467-025-62950-0

**Published:** 2025-09-03

**Authors:** Renyu Zheng, Mingfei Zhao, Jingshan S. Du, Tarunya Rao Sudarshan, Yicheng Zhou, Anant K. Paravastu, James J. De Yoreo, Andrew L. Ferguson, Chun-Long Chen

**Affiliations:** 1 Department of Chemical Engineering, University of Washington, Seattle, WA 98195 USA; 2 Physical Sciences Division, Pacific Northwest National Laboratory, Richland, WA 99352 USA; 3 Pritzker School of Molecular Engineering, University of Chicago, Chicago, IL 60637 USA; 4 School of Chemical and Biomolecular Engineering, Georgia Institute of Technology, Atlanta, GA 30332 USA; 5 Parker H. Petit Institute for Bioengineering and Biosciences, Georgia Institute of Technology, Atlanta, GA 30332 USA; 6 Department of Materials Science, University of Washington, Seattle, WA 98195 USA

**Keywords:** Self-assembly, Nanostructures, Polymers

Correction to: *Nature Communications* 10.1038/s41467-024-46839-y, published online 16 April 2024

In the version of the article initially published, Fig. 4d was wrong and incorrectly copied from Fig. 4b; the figure should have appeared as shown below. The correct version of the manuscript was reviewed, and the error was introduced after the peer review process was complete. The original article has been corrected.