# Supplementary material for: Assembly of short amphiphilic peptoids into nanohelices with controllable supramolecular chirality
Source: Nat Commun. 2024 Apr 16;15:3264. doi: 10.1038/s41467-024-46839-y (PMC11021492; doi:10.1038/s41467-024-46839-y)
Supplement: Supplementary file 1 — Supplementary Information [file 41467_2024_46839_MOESM1_ESM.pdf]

Supplementary Information

Table of Contents

1. **Supplementary methods**
   1.1 Materials
   1.2 Synthesis of peptoid oligomers
   1.3 Cleavage and purification of peptoid oligomers
   1.4 Self-assembly of peptoids into nanohelices
   1.5 Atomic force microscopy imaging
   1.6 Scanning/Transmission electron microscopy imaging
   1.7 X-ray powder diffraction
   1.8 Molecular Dynamics Simulation
   1.9 Theoretical model of ribbon width
2. **Supplementary discussion**
   2.1 Large-scale ADF-STEM image of peptoid nanohelices self-assembled at pH=7
   2.2 Large-scale AFM image of peptoid helices self-assembled at pH=7
   2.3 XRD data of Npm4Dig and Npm6Nce6
   2.4 Negative-stained ADF-STEM image of Npm4Dig nanohelices self-assembled at pH=4.
   2.5 AFM images of Npm4Dig nanohelices self-assembled at pH=4
   2.6 Amorphous aggregates of Npm4Dig self-assembled at pH=12
   2.7 Negatively-stained ADF-STEM image of left-handed Npm4-D-Ala helices
   2.8 Negatively-stained ADF-STEM image of right-handed Npm4-L-Ala helices
   2.9 AFM image of Npm4-D-Ala left-handed helices
   2.10 AFM image of Npm4-L-Ala right-handed helices
   2.11 XRD data of Npm4-L-Ala, Npm4-D-Ala, and Npm4Dig nanohelices.
   2.12 Solid-state C^{13} NMR data of nanohelices assembled from Npm4-L-Ala and Npm4-D-Ala
   2.13 Negatively stained TEM and AFM images of Npm4-L-Ala and Npm4NOH nanohelices

1. Supplementary methods

1.1 Materials

Rink amide resin (100-200 mesh) was purchased from Supra Science. Benzylamine (Npm, ≥98%), N, N′-diisopropyl carbodiimide (DIC, ≥99%), 4-Dimethylaminopyridine (DMAP, ≥98%), trifluoroacetic acid (TFA, ≥99%), and Fmoc-D-Ala-OH (≥98%) were purchased from Oakwood Products, Inc. Bromoacetic acid (BrAA, ≥98%) was purchased from Chem Impex, Inc. 4-Methylpiperidine (PIP, ≥98%) and diglycolic anhydride (Dig, ≥98%) were purchased from TCI America, Inc. Fmoc-Ala-OH (≥99%) and Fmoc-Gly-OH (≥99%) were purchased from Aapptec. Dimethylformamide (DMF, ≥99.5%), N-Methyl-2-pyrrolidone (NMP, ≥99%), Pyridine (≥99%), acetonitrile (ACN, ≥99.9%), and Dichloroethane (DCM, ≥99.9%) were from Sigma Aldrich, Inc. All of the above other reagents were purchased from commercial sources and used as received.

1.2 Synthesis of peptoid oligomers

The general synthesis procedure follows our protocol as previously reported[1,2]. In specific, rink amide resin (0.09 mmol) was used to synthesize C-terminal amphiphilic peptoids. In the synthesis procedure, the Fmoc groups on the resin were deprotected by adding 2 mL of 20% (v/v) PIP/DMF, agitating for 40 min, filtering, and washing with DMF. For all DMF washes, 1 mL DMF was added and then agitated for 30 seconds (repeated five times). An acylation reaction was then performed on the amino resin by adding 1.5 mL of 0.6 M BrAA in DMF and 0.30 mL of 50% (v/v) DIC/DMF. The mixture was agitated for 10 min at room temperature, filtered, and washed with DMF five times. Nucleophilic displacement of the bromine occurred by adding 1.5 mL of 0.6 M Npm in NMP, followed by agitation for 10 min at room temperature. The monomer solution was filtered from the resin and washed with DMF five times. The acylation and displacement steps were repeated four times until the targeted amphiphilic peptoid (Npm4) was synthesized.

To synthesize Npm4Dig, 200 mg of Diglycolic anhydride, 0.016 g DMAP, 1 mL pyridine, and 2.5 mL DCM were added to Npm4 resin. The mixture was agitated at room temperature overnight, filtered, and washed with DCM 5 times.

To synthesize Npm4-L/D-Ala, 300 mg of Fmoc-Gly-OH, 1mL DIC, and 1mL DMF were added to Npm4 resin and agitated at room temperature overnight. The mixture was filtered and washed with DMF 5 times. Then, the Fmoc groups were deprotected by adding 2 mL of 20% (v/v) PIP/DMF. After that, 300 mg of Fmoc-D-Ala-OH or Fmoc-L-Ala-OH, 1 mL DIC, and 1 mL DMF were added to the mixture, agitating at room temperature overnight, filtered, washed, and deprotected by adding 2 mL of 20% (v/v) PIP/DMF. The resultant mixtures were filtered and washed 5 times with DMF and then 5 times with DCM.

1.3 Cleavage and purification of peptoid oligomers

The crude product was cleaved from the resin by adding 95% trifluoroacetic acid (TFA) in water, which was then evaporated under vacuum with a Biotage V-10 evaporation system. Finally, crude peptoids were dissolved in H2O/CH3CN (v/v = 1:1) for HPLC purification. The crude

products were purified by reverse-phase HPLC on an XBridge Prep C18 10 µm OBDTM (10 µm, 19mm × 100 mm), using an adaptable acetonitrile gradient in H2O with 0.1% TFA over 15 min. Purified peptoids were analyzed using Waters ACQUITY reverse phase UPLC (corresponding gradient at 0.4 mL/min over 7 min at 40 °C with an ACQUITYBEH C18, 1.7 µm, 2.1 mm× 50mm column) that was connected with a Waters SQD2 mass spectrometry system. The final peptoid product was lyophilized twice from its solution in a mixture (v/v = 1:1) of water and acetonitrile. The peptoid powder was finally divided into small portions (1.0×10^{-6} mol) and stored at room temperature.

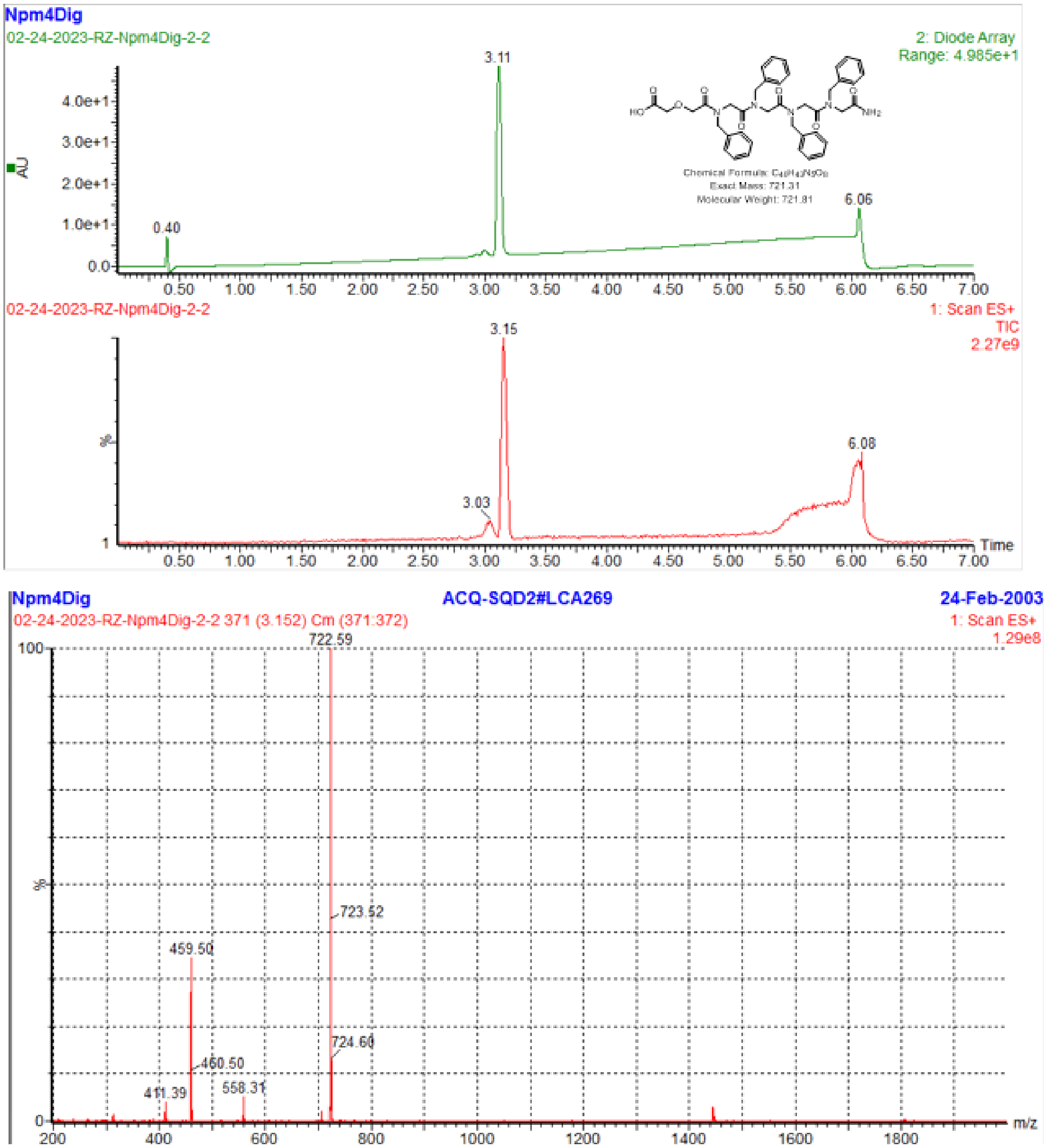

Supplementary Figure 1 | UPLC-MS data of HPLC-purified peptoid: Npm4Dig; the insert is the chemical structure of this peptoid

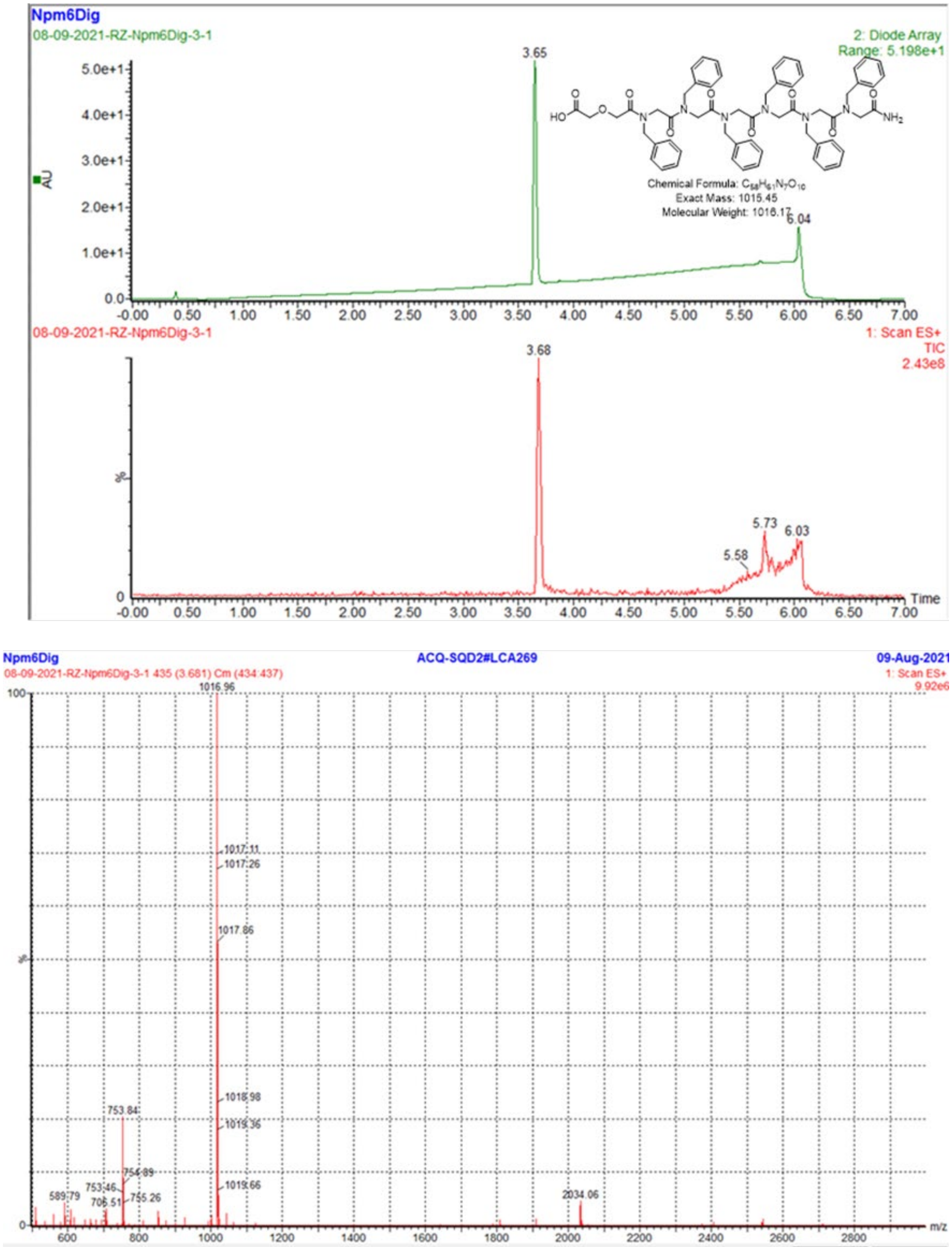

Supplementary Figure 2 | UPLC-MS data of HPLC-purified peptoid: Npm6Dig; the insert is the chemical structure of this peptoid

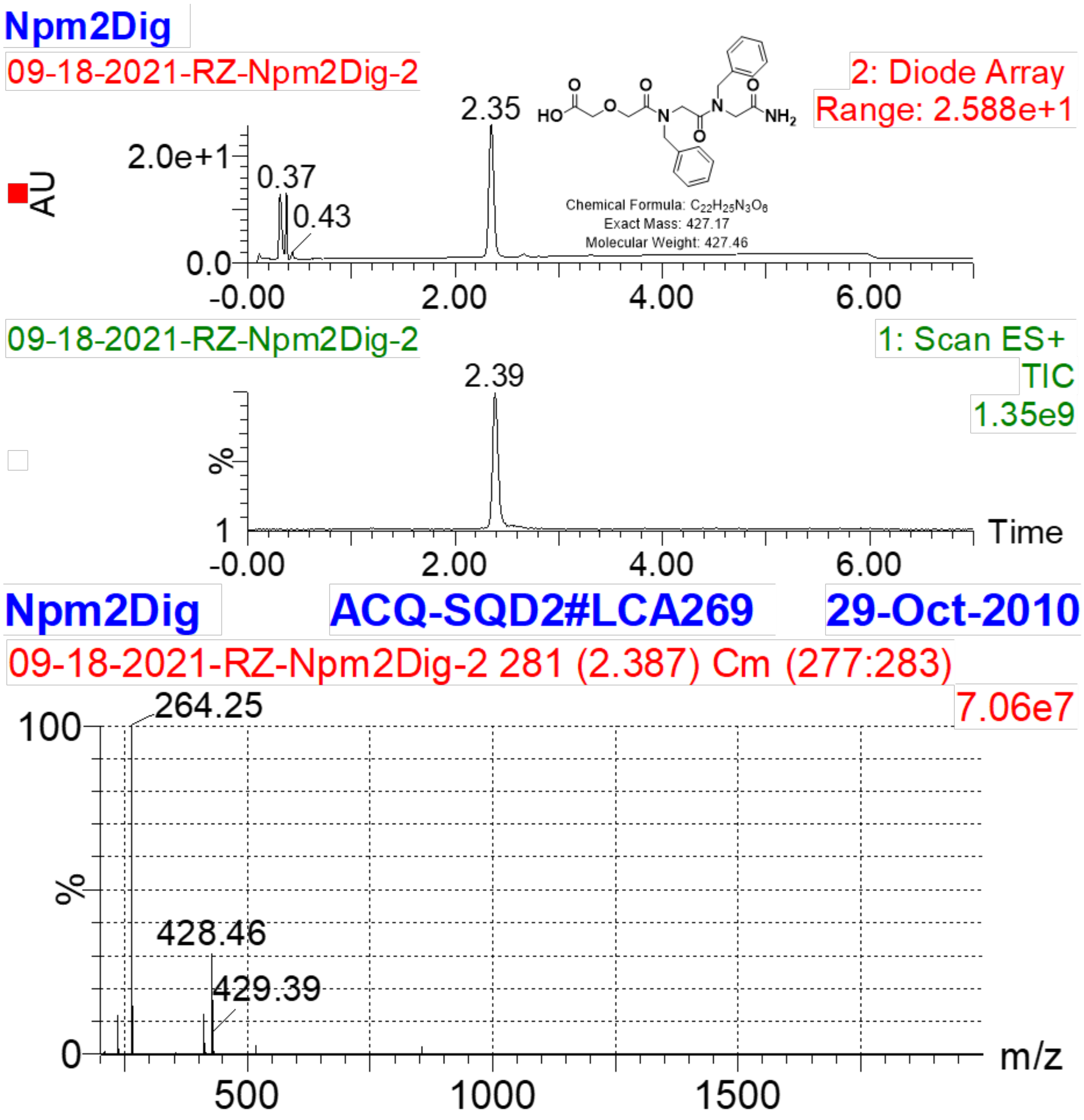

Supplementary Figure 3 | UPLC-MS data of HPLC-purified peptoid: Npm2Dig; the insert is the chemical structure of this peptoid

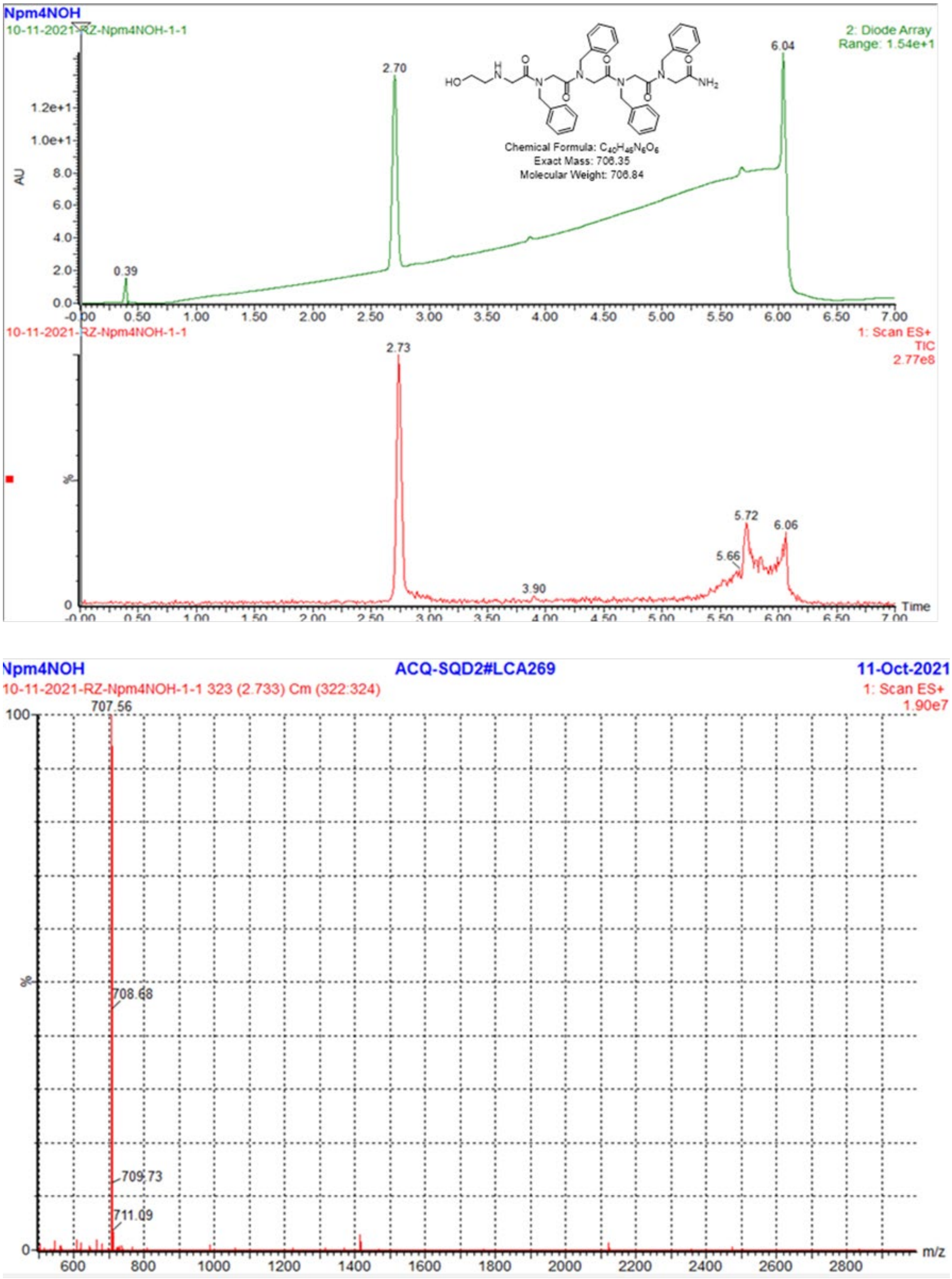

Supplementary Figure 4 | UPLC-MS data of HPLC-purified peptoid: Npm4Noh; the insert is the chemical structure of this peptoid

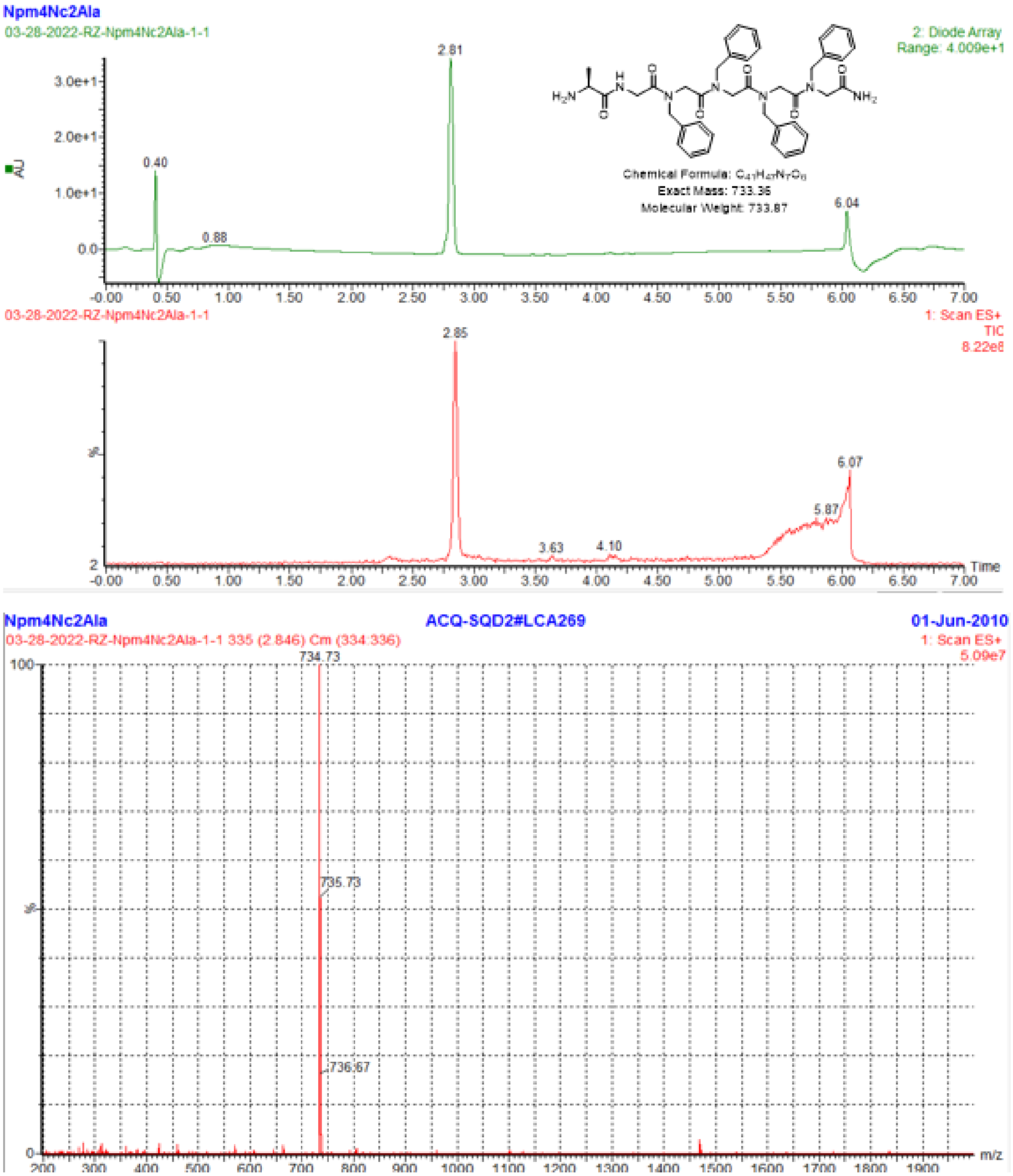

Supplementary Figure 5 | UPLC-MS data of HPLC-purified peptoid: Npm4-L-Ala; the insert is the chemical structure of this peptoid.

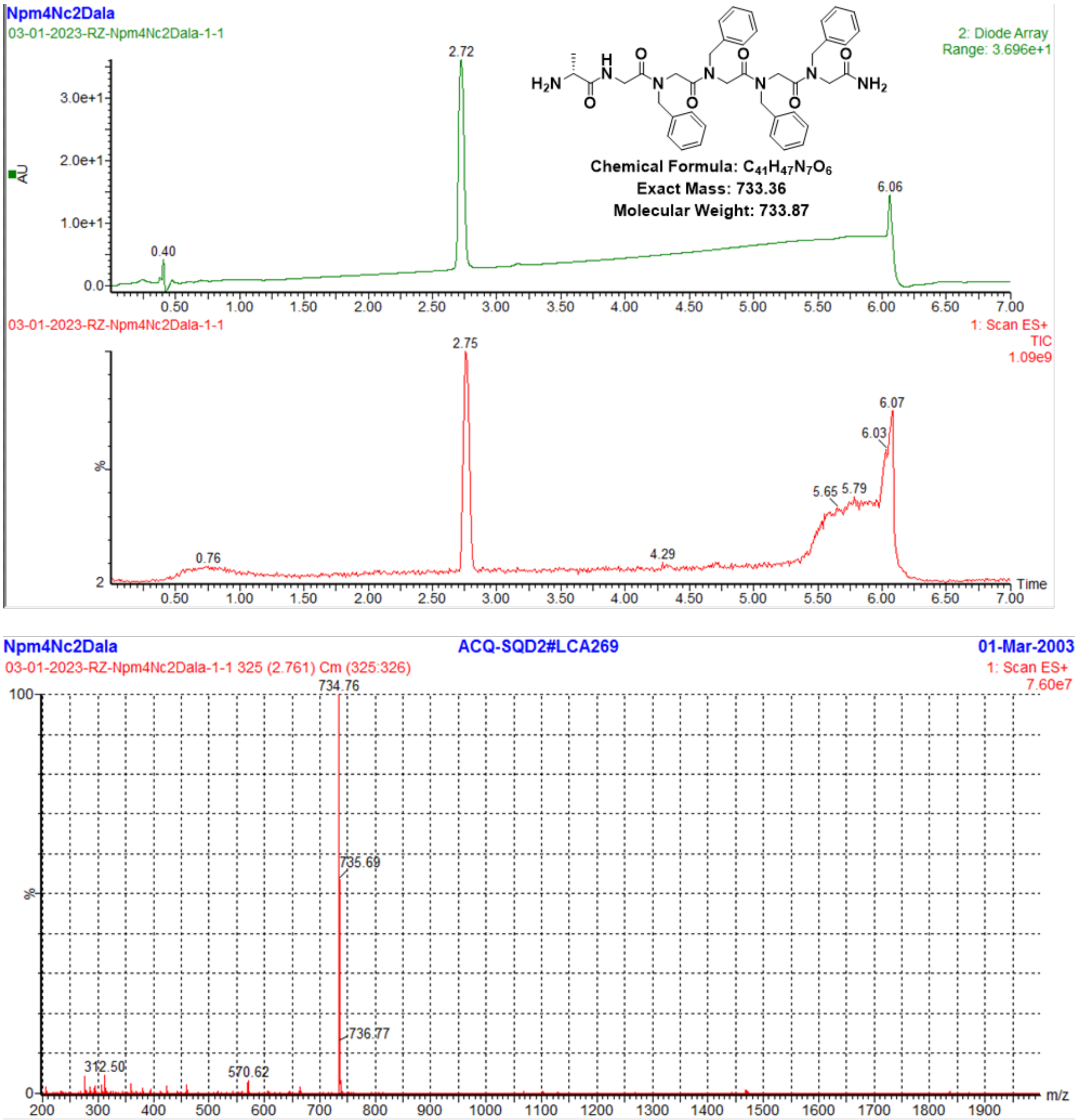

Supplementary Figure 6 | UPLC-MS data of HPLC-purified peptoid: Npm4-D-Ala; the insert is the chemical structure of this peptoid.

1.4 Self-assembly of peptoids into nanohelices

1 µmol of lyophilized peptoid powders were dissolved in 200 µL of water and acetonitrile (v/v = 1:1) mixture to make a 5.0 mM clear solution, with pH around 4. The mixture was then put in the 4 °C refrigerator for slow evaporation. A gel-like material consisting of nanohelices was obtained after 2 to 3 days.

For the pH adjustment of the self-assembly, droplets of 1 M and NaOH/HCl aqueous solution were added to 5.0 mM peptoid ACN/H_2O solution until the desired pH was obtained. The resultant solutions were then used for slow evaporation at 4 °C.

1.5 Atomic force microscopy imaging

Ex-situ (atmospheric) AFM was performed on a Bruker MultiMode 8 in tapping mode with Bruker ScanAsyst-Air probes (k = 0.4 N/m) at room temperature. A 2 µL drop of the gel-like assembly solution was diluted with 40 µL deionized water and placed onto a freshly cleaved mica substrate for 5 min. The solution was then dried with filter paper and N_2 flow.

1.6 Scanning Transmission electron microscopy (STEM)

STEM was performed on an FEI Tecnai operating at an accelerating voltage of 200 keV for annular dark-field (ADF) imaging. To prepare STEM samples, a 2 µL drop of the assembly solution was diluted in 10 µL of deionized water and drop-cast onto carbon-coated copper grids, and excess liquids were removed with filter paper after 10 min. For negative staining, 5 µL of phosphotungstic acid (wt 2%) was dropped onto the TEM grid, and excess liquids were removed with filter paper after 2 min.

1.7 X-ray powder diffraction (XRD)

Powder XRD samples were prepared by lyophilization of the self-assembled solution. Powder XRD data were collected at a multiple-wavelength anomalous diffraction and monochromatic macromolecular crystallography beamline, 8.3.1, at the Advanced Light Source at Lawrence Berkeley National Laboratory. Beamline 8.3.1 has a 5T single pole superband source with an energy range of 5–17 keV. Data were collected with a 3 × 3 CCD array (ADSC Q315r) detector at a wavelength of 1.1159 Å. Datasets were collected with the detector 200 mm from the sample. PNT suspensions or pellets were pipetted onto a Kapton mesh (MiTeGen) and dried. All XRD data were processed with custom Python scripts.

1.8 Molecular dynamics simulation

The short amphiphilic diblock peptoid investigated in this work, Npm4Dig, consists of four hydrophobic N-phenylmethyl glycine residues (Npm) and one polar diglycolic acid (CH2OCH3COOH) tail (Dig). The peptoid structure is generated in Avogadro 1.2.0.[3] The Npm4 block is modeled using the improved all-atom CGenFF peptoid force field developed by Weiser and Santiso as a peptoid-tuned modification of the CHARMM22 peptide force field.[4-6] The Dig tail is adapted from MOAC residue (methoxyacetic acid) from all-atom CGenFF force field.[5,6] The fibrils simulated in this work start with the terminal bilayer nanosheets with different protonation states. The formal pKa of the carboxylic acid group present on the Dig tail is ~3.5.[7] Simulations are therefore conducted with Npm4Dig peptoids with protonated -COOH tails corresponding to low-pH conditions. Simulations conducted with Npm4Dig peptoids with deprotonated -COO^- tails indicated that the peptoid stack would rapidly disassemble due to electrostatic repulsion between the charged -COO^- groups. The precise pH range over the tails expected to be protonated is uncertain due to potential changes in the pKa of the carboxylic acid group due to intramolecular effects and also changes in the local environment due to aggregation into a peptoid stack and exclusion of water solvent within the aggregate. The TIP3P

model was used to simulate water.[8] The initial configurations of the system was built from Packmol as shown in Fig.2a.[9] All-atom molecular dynamics simulations were conducted in Gromacs 2019.2.[10] Molecular trajectories were visualized in VMD.[11]

Initially, we built up monolayer Npm4Dig nanofibrils using the previous scheme we developed for other longer peptoid sequences.[12] However, the monolayer Npm4Dig fibrils are not stable in the pure water environment, which is likely due to the shorter hydrophobic blocks in Npm4Dig peptoids leading to large exposure area of hydrophobic blocks and weaker π-stacking interactions. As a result, the bilayer packing structure as shown in Fig. 2a was developed comprising two layers of Npm4Dig peptoids with hydrophilic Dig tails pointing towards water. There are 6 columns in each layer with inter-column distances of 1.35 nm. In each column, there are 40 monomers with separations of 45 nm. Nanofibrils were placed in a (30 × 10 × 30) nm^3 box and solvated with 50,250 water molecules at a density of ∼1 g/cm^3.

The system was relaxed by first applying steepest descent energy minimization to remove forces in excess of 1000 kJ/(mol·nm). Initial atom velocities were assigned from a Maxwell-Boltzmann distribution at 300 K. Periodic boundary conditions were applied in all three dimensions. Systems were first equilibrated for 400 ps in the NPT (i.e., isothermal-isobaric) ensemble at 300 K and 1 bar employing a velocity rescaling thermostat[13] with a time constant of 0.1 ps and a Berendsen barostat[14] with a time constant of 1.0 ps and compressibility of $4.5{\times}10^{-5}$ bar^{-1}. We conducted production runs of 20 ns in the NPT ensemble at 300 K and 1 bar employing a Nosé-Hoover thermostat [15] with a time constant of 1.0 ps and Parrinello-Rahman barostat[16] with a time constant of 1.0 ps and compressibility of $4.5{\times}10^{-5}$ bar^{-1}. Four independent simulations were conducted starting from the same initial structure but different initial velocity distributions.

The twisted dihedrals in Fig.2c of the main text are defined by the angle (θ) between the upper edge vector ($\vec{v}_u$) and the upper edge vector ($\vec{v}_l$), i.e., $\vec{v}_u \cdot \vec{v}_l = |\vec{v}_u||\vec{v}_l| \cos\theta$. $\vec{v}_u$ and $\vec{v}_l$ are calculated from center-of-mass (COM) positions of the monomers at two boundary layers of the fibril as indicated in yellow in the upper image of Fig.2a. In detail, using the *Line.bestfit* function from the *skspatial* python package,[17] two unit direction vectors representing the upper and lower edges of fibrils can be calculated by fitting upper and lower edges of COM points respectively through the command "*line_lower.direction*". The error bars in Fig.2c are the standard deviations calculated from four independent runs.

1.9 Theoretical model of ribbon width

To gain insight and understanding of the twisted ribbon geometry as a function of the solvent conditions and intermolecular attractions, we developed a simple phenomenological free energy model for the self-assembly of the twisted ribbons. The model is an adaptation of that originally developed by Boden and coworkers for twisted β-sheet stacks of peptides[18,19] and subsequently sophisticated by Rüter et al.[20]. The present model comprises a favorable hydrophobic association of the peptoids driven by interfacial free energy that promotes the formation of wide ribbons, and an energetic penalty for poorer intermolecular axial stacking between adjacent peptoids with increasing ribbon width that favors thin ribbons. The balance of

these two contributions provides a molecular basis for the observed increase in ribbon width with lowering pH and offers molecular insight into the self-assembled geometry of the twisted peptoid ribbons.

We construct the model based on an idealized geometry of a twisted peptoid ribbon illustrated in Fig. S7. Treating each peptoid as a stiff rod, the $N = 2M$ peptoids comprising a single layer of the twisted ribbon are considered to lie in the plane with each peptoid assigned an index m according to its distance from the central axis (Fig. S7a). Each layer in the ribbon is offset from those above and below it by a planar angle θ that mediates the overall twist (Fig. S7b). We denote the breadth of the ribbon as $2l$. The spacing between peptoids in the plane is denoted d. The width of the ribbon containing N=2M peptoids is then $D=d(N-1)=d(2M-1)$. The radial distance of a peptoid with index m from the central axis of the ribbon is $r_m = ½d_m = d(|m|-½)$. The arclength between peptoids of index $|m|$ in successive layers of the twisted ribbon stack is $a = ½d_m\,\theta$. The spacing between successive layers of the twisted ribbon is δ and the pitch – the axial distance over which the twisted ribbon executes a complete turn – is λ (Fig. S7c). The number of layers constituting a complete pitch is $P_\lambda=\lambda/\delta$ and the number of layers in a ribbon of length L is $P=L/\delta$. Simple geometric considerations dictate that the linear through-space distance δ_c of the centers of mass of the two peptoids of index m in successive layers of the twisted ribbon is given by $\delta_c^2 = \delta^2 + (½d_m\theta)^2$, where we have assumed that the twist angle is sufficiently small that the arclength is a good approximation for the chord (Fig. S7d).

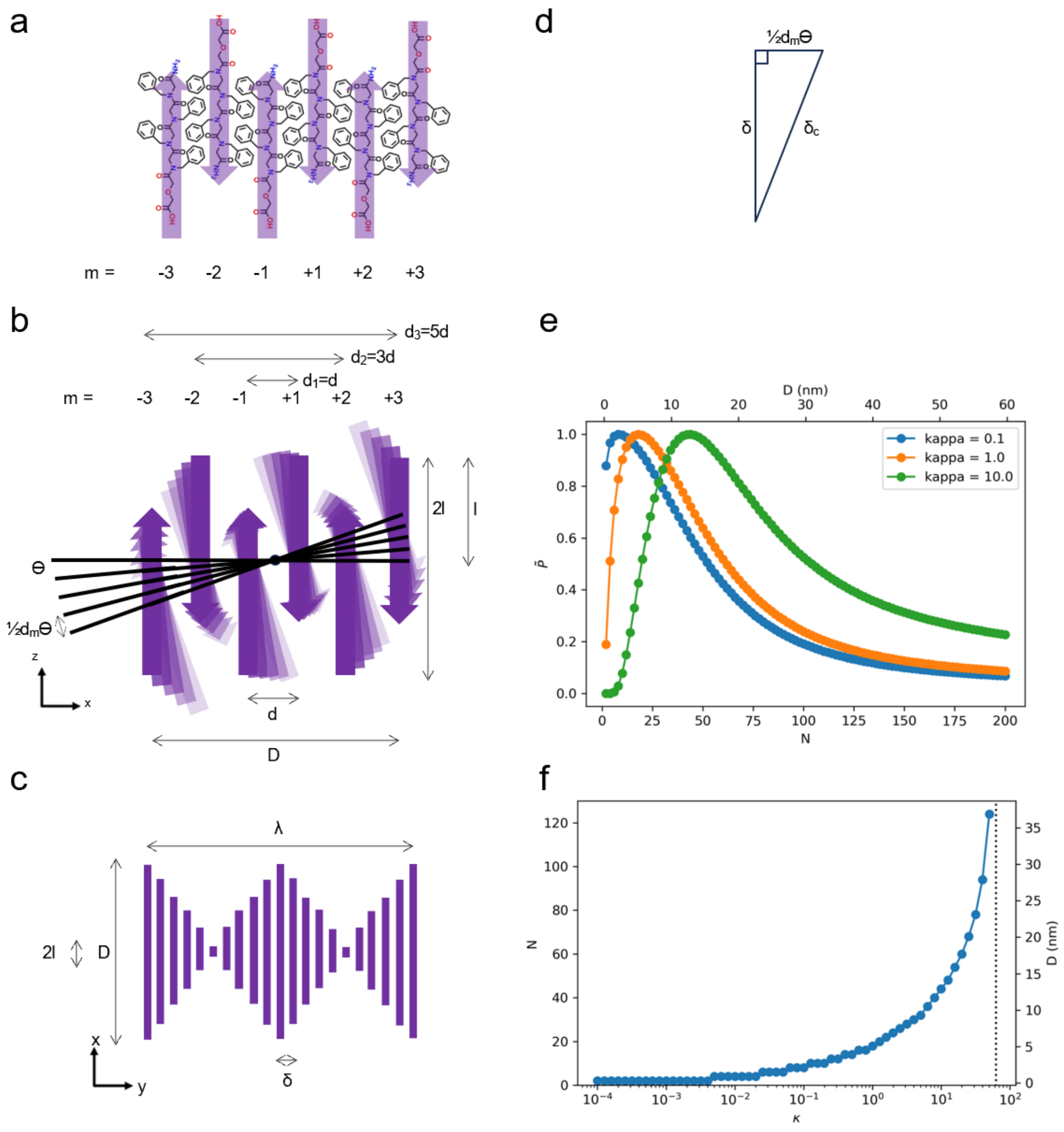

Supplementary Figure 7 | . Schematic illustration of idealized twisted ribbon geometry employed in the theoretical model of peptoid ribbon energy and predictions of the thermodynamically favored ribbon width. (a) Illustration of a single layer of a peptoid ribbon six molecules wide (cf. Fig. 1b). Each peptoid is represented schematically as a directed arrow and assigned an index *m* where there are $N = 2M$ peptoids in a single layer of the ribbon. (b) Illustration of a top-down view of an idealized twisted peptoid ribbon. Each layer of the ribbon is rotated by a planar angle θ relative to the layer above. The breadth of the ribbon – corresponding to the z-dimension of the nearest layer of the ribbon illustrated here – is 2*l*. The width of the ribbon – corresponding to the x-dimension – is *D*, where the linear spacing between peptoids is *d*. The width of a ribbon containing $N = 2M$ peptoids per layer is $D = d_M = d(2M\text{-}1) = d(N\text{-}1)$. The radial distance of a peptoid with index |*m*| from the axial center of the ribbon is $r_m = ½d_m = ½d(2|m|\text{-}1) = d(|m|\text{-}½)$, where the modulus |·| indicates the absolute value. The arclength between peptoids of index |*m*| in successive layers of the twisted ribbon stack is $a = ½d_m\,\theta$. (c) Schematic illustration of a side-on view of an idealized twisted peptoid ribbon. As the

ribbon twists around its axis in the y-dimension, it possesses a maximum extent of D and a minimum extent of $2l$. The spacing between successive layers of the ribbon is δ. The pitch of the ribbon – the axial distance over which it executes a complete turn – is λ. (d) The center of mass distance between two peptoids with index m in successive layers of the twisted ribbon is given by simple geometric considerations. The axial distance between successive peptoid layers is δ. The distance between the centers of mass in the perpendicular (i.e., radial) plane under the action of the twist is given by the chord $c = 2r_m.\sin(½θ) = d_m.\sin(½θ)$ of the corresponding arc of the circle of radius $r_m = ½d_m$ subtended by the angle θ. For small θ, the chord is well approximated by the arclength under the small θ expansion of the sine function (i.e., $\sin(x) \approx x$) such that $c \approx a = ½d_mθ$. The linear through-space distance $δ_c$ of the centers of mass of the two peptoids of index m in successive layers of the twisted ribbon is then given by the Pythagorean theorem as $δ_c^2 = δ^2 + (½d_mθ)^2$. (e) Relative probabilities of ribbons of various widths D and peptoids per layer N as a function of the dimensionless parameter κ specifying the relative strength of the interfacial and van der Waals interactions, where $κ = e_{interfacial}/e_{dispersion} = γlδ/ε$. Without loss of generality, the curves are scaled such that the maximum relative probability is $\tilde{P} = 1$. The most probable width of the ribbon increases with increasing κ as the interfacial tension makes it more favorable to construct wider ribbons and pay the penalty of poorer van der Waals stacking interactions at large radial distances from the ribbon axis in service of reducing the unfavorable interfacial interaction of the exposed edges of the peptoid ribbon with the solvent. The most probable ribbon widths and number of peptoids per layer predicted by the model for κ = [0.1, 1.0, 10] are D = [2.1, 5.1, 12.9] nm and N = [8, 18, 44]. (f) The number of peptoids per layer and ribbon width of the most probable ribbon geometry predicted by the model as a function of κ. For small κ ($κ \lesssim 0.005$), the thinnest ribbon width of $N = 2$ (i.e., $M = 1$) permitted by the model is predicted to be the most probable. As κ increases, the model predicts an increase in the width of the most probably ribbon and a divergence towards infinite ribbon width (i.e., $N = \infty$) at κ = 62.5 (vertical dashed line).

Based on these geometric concerns, we now proceed to construct the phenomenological free energy model. We consider two contributions to the free energy of the ribbon: interfacial tension and packing dependent dispersion interactions.

Interfacial Tension. The first contribution is the free energy due to interfacial tension associated with the exposed hydrophobic faces of the peptoids comprising the outer edge of the ribbon (i.e., those with index $|m|=M$). A primary driving force for assembly of these ribbons is the hydrophobic effect that promotes assembly of the peptoids to bury their hydrophobic groups from the water solvent. Ignoring contributions from the exposed upper and lower edge of the ribbon – a good assumption for high-aspect ratio ribbons where the length L is much larger than the width D – the interfacial free energy contribution for a ribbon of length L and width $D = d_M = d(2M\text{-}1) = d(N\text{-}1)$ can be expressed as,

$$U_{\text{interfacial}} = 2\gamma A_{\text{face}} = 2\gamma(2lL) = 2\gamma(2l\delta P) = 4\gamma l\delta P, \qquad \text{Eqn. S1}$$

where $A_{\text{face}} = 2lL$ is the exposed surface area of the two solvent-exposed edges of the ribbon and $P = L/\delta$ is the number of stacked planes in the ribbon of length L. The free energy per peptoid monomer follows by dividing the energy of the ribbon by the total number of peptoids comprising the ribbon,

$$u_{\text{interfacial}}(M) = {U_{\text{interfacial}}}/{2MP} = {2\gamma l\delta}/{M}. \qquad \text{Eqn. S2}$$

Dispersion Interactions. The second contribution to the ribbon free energy is the free energy associated with favorable dispersion interactions between the rotationally offset layers of peptoids constituting the twisted ribbon. We assume peptoids stack in-register such that we model the interaction of a peptoid with index *m* with the corresponding peptoids of index *m* in the layers immediately above and below it. We model the favorable dispersion interactions as the attractive part of the Lennard-Jones potential such that the dispersion energy experienced by a peptoid with index *m* is,

$$\begin{aligned} e_{\text{dispersion}}(m) &= -{2\varepsilon_H}/{\delta_c^6} \\ &= -{8\varepsilon\lambda^6}/{\left[\lambda^2 + \left[2\pi d\left(m-\tfrac{1}{2}\right)\right]^2\right]^3}, \end{aligned} \qquad \text{Eqn. S3}$$

where $\varepsilon_H = 4\varepsilon\sigma^6$ is the Hamaker constant characteristic of the peptoid-peptoid interactions, which we have related to the Lennard-Jones energy ε and size σ parameters, δ_c is the through space linear distance between two peptoids and for peptoids of index *m* is given by $\delta_c^2 = \delta^2 + (\tfrac{1}{2} d_m \theta)^2$ (Fig. S4d) and $d_m = 2d(|m|-\tfrac{1}{2})$ (Fig. S4b), and the factor of two accounts for the dispersion interactions with the peptoid of index *m* in the layer above and in the layer below. We further choose to adopt the inter-layer spacing as a characteristic interaction distance and set σ=δ such that $\varepsilon_H = 4\varepsilon\delta^6$.

Now, summing over all such dispersion interactions within a single stack of the ribbon, we obtain,

$$\begin{aligned} U_{\text{dispersion}}(M) &= \tfrac{1}{2}\textstyle\sum_{m=-M, m\neq 0}^{+M} e_{\text{dispersion}}(m) \\ &= \textstyle\sum_{m=1}^{M} e_{\text{dispersion}}(m) \\ &= -8\varepsilon\lambda^6 \textstyle\sum_{m=1}^{M} \left[\lambda^2 + \left[2\pi d\left(m-\tfrac{1}{2}\right)\right]^2\right]^{-3}, \end{aligned} \qquad \text{Eqn. S4}$$

where the factor of one-half corrects for double counting of the dispersion interactions within the sum. The dispersion energy per peptoid monomer follows by dividing through by the number of peptoids comprising a single layer of the stack,

$$\begin{aligned} u_{\text{dispersion}}(M) &= {U_{\text{dispersion}}(M)}/{2M} \\ &= -{4\varepsilon\lambda^6}/{M} \textstyle\sum_{m=1}^{M} \left[\lambda^2 + \left[2\pi d\left(m-\tfrac{1}{2}\right)\right]^2\right]^{-3}. \end{aligned} \qquad \text{Eqn. S5}$$

Total Free Energy. The overall free energy per peptoid monomer within a twisted ribbon of width *N* = 2*M* is given by summing these two contributions,

$$\begin{aligned} u(M) &= u_{\text{interfacial}}(M) + u_{\text{dispersion}}(M) \\ &= {2\gamma l\delta}/{M} - {4\varepsilon\lambda^6}/{M} \textstyle\sum_{m=1}^{M} \left[\lambda^2 + \left[2\pi d\left(m-\tfrac{1}{2}\right)\right]^2\right]^{-3} \end{aligned}$$

Finally, it is convenient to work in a dimensionless gauge by dividing the energy through by the Lennard-Jones energy parameter,

$$\begin{aligned}\tilde{u}(M) &= {}^{u(M)}/_{\varepsilon} \\ &= {}^{2}/_{M}\left({}^{\gamma l\delta}/_{\varepsilon}\right) - {}^{4\lambda^6}/_{M}\sum_{m=1}^{M}\left[\lambda^2 + \left[2\pi d\left(m-\tfrac{1}{2}\right)\right]^2\right]^{-3} \\ &= {}^{2\kappa}/_{M} - {}^{4\lambda^6}/_{M}\sum_{m=1}^{M}\left[\lambda^2 + \left[2\pi d\left(m-\tfrac{1}{2}\right)\right]^2\right]^{-3} \\ &= {}^{1}/_{M}\left[2\kappa - S(M;\lambda,d)\right],\end{aligned} \qquad \text{Eqn. S6}$$

where the first term is the dimensionless interfacial free energy contribution and the second term is the dimensionless dispersion energy contribution. In going from the second line to the third, we have defined a dimensionless parameter κ as the ratio of the characteristic magnitudes of the interfacial and dispersion free energy contributions,

$$\kappa = {}^{\gamma l\delta}/_{\varepsilon}, \qquad \text{Eqn. S7}$$

and in going from the third line to the fourth, we defined the summation accumulating the dimensionless dispersion energy contributions as,

$$S(M;\lambda,d) = 4\lambda^6\sum_{m=1}^{M}\left[\lambda^2 + \left[2\pi d\left(m-\tfrac{1}{2}\right)\right]^2\right]^{-3}, \qquad \text{Eqn. S8}$$

where we explicitly indicate the functional dependence on *M* and express λ and *d* as parameters. The expression in Eqn. S6 presents a simple prescription for identifying the most probable ribbon as that with the number of monomers per layer *N*=2*M* that minimizes the dimensionless free energy $\tilde{u}(M)$ and where the probability of a ribbon is proportional to the Boltzmann factor of the free energy $\tilde{P}(M) \propto \exp\left(-\tilde{u}(M)\right)$.

Model Parameters. The governing equation specifying the preferred ribbon width *M* contains only three parameters: (i) the pitch of the ribbon λ, (ii) the spacing between peptoids in a layer *d*, and (iii) the lumped dimensionless parameter specifying the ratio of the interfacial and dispersion free energy contributions $\kappa = e_{\text{interfacial}}/e_{\text{dispersion}} = \gamma l\delta/\varepsilon$. For the peptoids in this work, we adopt characteristic values of λ = 100 nm and *d* = 0.3 nm motivated by the molecular geometry of the peptoids and the experimentally observed twist in the ribbon (cf. Figs. 1 and 3). With regards to κ, we adopt γ = 2.4×10^{-20} J/nm^2 as a characteristic value of the surface tension appropriate for hydrophobic hydrocarbons= groups,[20,21] ε = 6.95×10^{-21} J = 1 kcal/mol as a characteristic value for pi-stacking dispersion interactions that have been reported to lie in the range 0.7-3 kcal/mol for polystyrene and benzene,[22] l = 1 nm is a characteristic length scale for the peptoid monomers, and δ = 0.3 nm is a characteristic length scale for the inter-layer spacing in the stacked ribbon. This produces a characteristic value of κ = 1.036. The specific value of κ is less important than its magnitude. The primary value of the model is in predicting how the thermodynamically preferred ribbon width changes as a function of κ. Increasing κ corresponds to an elevation of the surface tension relative to the dispersion interactions and can be achieved by, for example, lowering the pH to push the chemical equilibrium of the acidic groups from the COO^- to the COOH form to elevate the hydrophobicity of the peptoid monomers. Decreasing κ

corresponds to a reduction of the surface tension relative to the dispersion interactions by increasing the hydrophilicity, and can, conversely, be achieved by raising the pH.

We present in Fig. S7e the relative probabilities $\tilde{P}(M) \propto \exp\left(-\tilde{u}(M)\right)$ of ribbons comprising different numbers of monomers per layer N=2M, and therefore different widths $D = d_M = d(2M-1) = d(N-1)$, resulting from numerical evaluation of Eqn. S6 for κ = 0.1, 1.0, and 10. We present in Fig. S4f a plot of the most preferred ribbon width (i.e., the maxima of the curves in Fig. S7e) as a function of κ. These plots illustrate that the model predicts an increasing width of ribbons with increasing κ. Physically, an elevated interfacial tension makes it more favorable to construct wider ribbons and pay the penalty of poorer van der Waals stacking interactions at large radial distances from the ribbon axis in service of reducing the unfavorable interfacial interaction of the exposed edges of the peptoid ribbon with the solvent. The trends predicted by the model are in good agreement with the increase in experimentally observed ribbon width from (5.2 ± 0.4) nm at pH 7 to (15.6 ± 2.42) nm at pH 4 corresponding to an elevation in κ.

Singularity Analysis. As illustrated in Fig. S7f, the numerical solution of Eqn. S6 for the value M that minimizes $\tilde{u}(M)$ exhibits and apparent singularity at κ ≈ 62.5. We can understand the origin of this behavior by taking the first derivative of $\tilde{u}(M)$ and setting it to zero to develop an implicit expression for M* defining the value of M for which $\tilde{u}(M)$ possesses a stationary point,

$$d\tilde{u}(M)/_{dM} = {-S'(M;\lambda,d)}/_{M} - {2\kappa}/_{M^2} + {S(M;\lambda,d)}/_{M^2} = 0,$$

$$\Rightarrow M^* = {[S(M^*;\lambda,d) - 2\kappa]}/_{S'(M^*;\lambda,d)}, \qquad \text{Eqn. S9}$$

where $S'(M;\lambda,d)$ denotes the M derivative of $S(M;\lambda,d)$. We make progress by observing that $S(M;\lambda,d)$ is a non-decreasing function of M (Eqn. S8), and, as a corollary, $S'(M;\lambda,d)$ is non-negative. Numerical continuation and extrapolation of the sum $S(M;\lambda,d) = 4\lambda^6 \sum_{m=1}^{M} \left[\lambda^2 + \left[2\pi d\left(m - \frac{1}{2}\right)\right]^2\right]^{-3}$ indicates that $S(M \to \infty;\lambda,d) \to 125$. In light of these observations, inspection of Eqn. S9 reveals that a self-consistent solution for M* can only exist on the positive domain (i.e., 0 < M* < ∞) for a positive right-hand side numerator such that $S(M;\lambda,d) - 2\kappa > 0 \Rightarrow \kappa < \frac{1}{2}S(M;\lambda,d)$. It follows, therefore, that there is a critical value of $\kappa_{\text{critical}} = \frac{1}{2}S(M \to \infty;\lambda,d) = {125}/_{2} = 62.5$, beyond which no positive solutions for M* are available. This coincides with the numerically observed divergence of M* in Fig. S7f.

1.10 NMR experimental description

Samples for solid state NMR experiments were prepared by dissolving 1 µmol of lyophilized peptoid powders in 100 µL of water and acetonitrile (v/v = 1:1) mixture to make a 10.0 mM clear solution, with pH around 4. The samples were then self-assembled as described above (section 1.4). These samples were then ultracentrifuged for 40 minutes into Bruker 3.2 mm NMR rotors at 4 °C at 150,000 RCF with Ultra-clear tubes in a SW-41 Ti swinging bucket rotor fitted onto a Beckman Optima XPN-100 centrifuge. ^{13}C cross-polarization magic angle spinning (CPMAS) spectra were collected on a 11.75 T magnet with 500 MHz ^{1}H NMR frequency. A 3.2mm Bruker

Low-E 1H/13C/15N NMR probe was used in a Bruker spectrometer. A 12.5 kHz magic angle spinning speed was used for all samples. Signals were averaged over approximately 13 hours.

2. Supplementary discussion

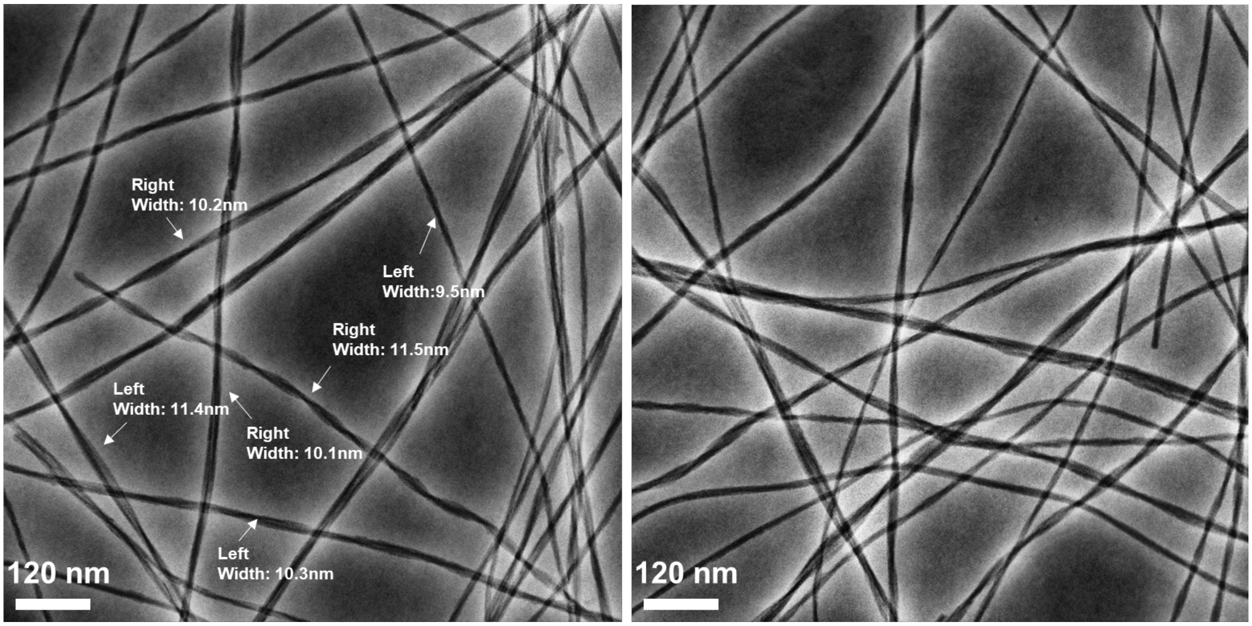

Supplementary Figure 8 | Negatively stained ADF-STEM images of Npm4Dig nanohelices self-assembled at pH = 7. The ratio of left- and right-hand twists is close to 1:1.

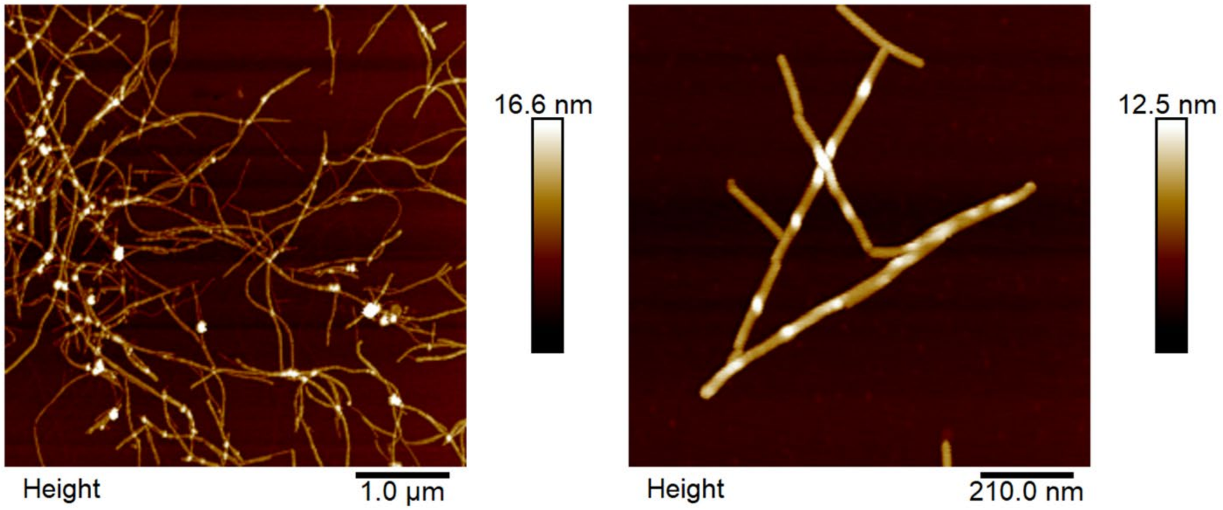

Supplementary Figure 9 | AFM images of Npm4Dig nanohelices self-assembled at pH = 7. Height distribution shown in Figure 3d is calculated from these nanohelices. The height distribution of these nanohelices is 5.2 ± 0.4 nm based on 30 measurements of nanohelices.

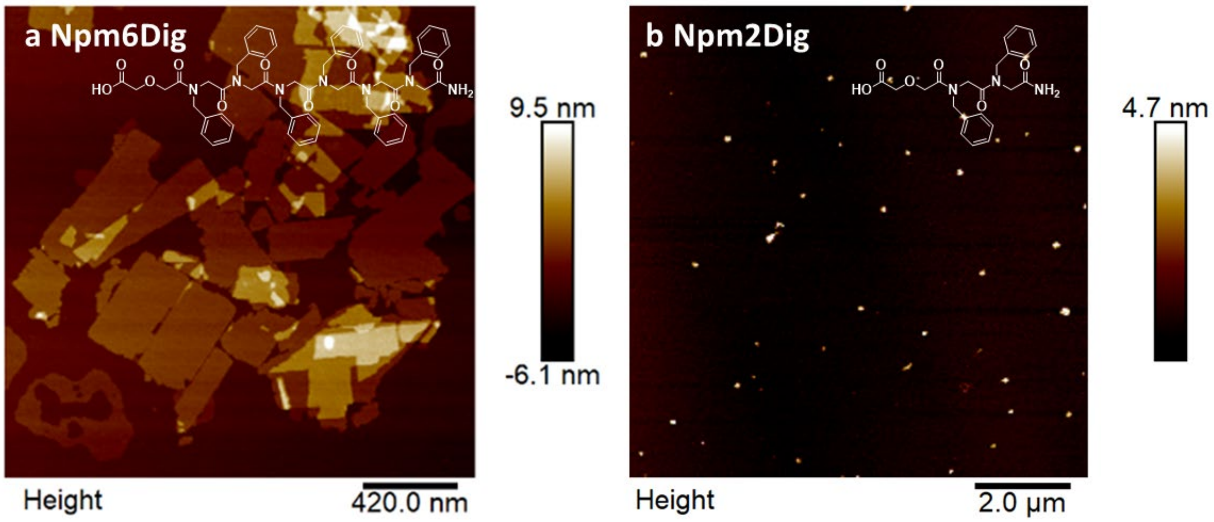

Supplementary Figure 10 | AFM images of peptoid assemblies formed from assembling peptoids with same polar domain but different hydrophobic domain with varied numbers of Npm groups. a) AFM image of nanosheets self-assembled from Npm6Dig. **b**) AFM image of isolated particles self-assembled from Npm2Dig.

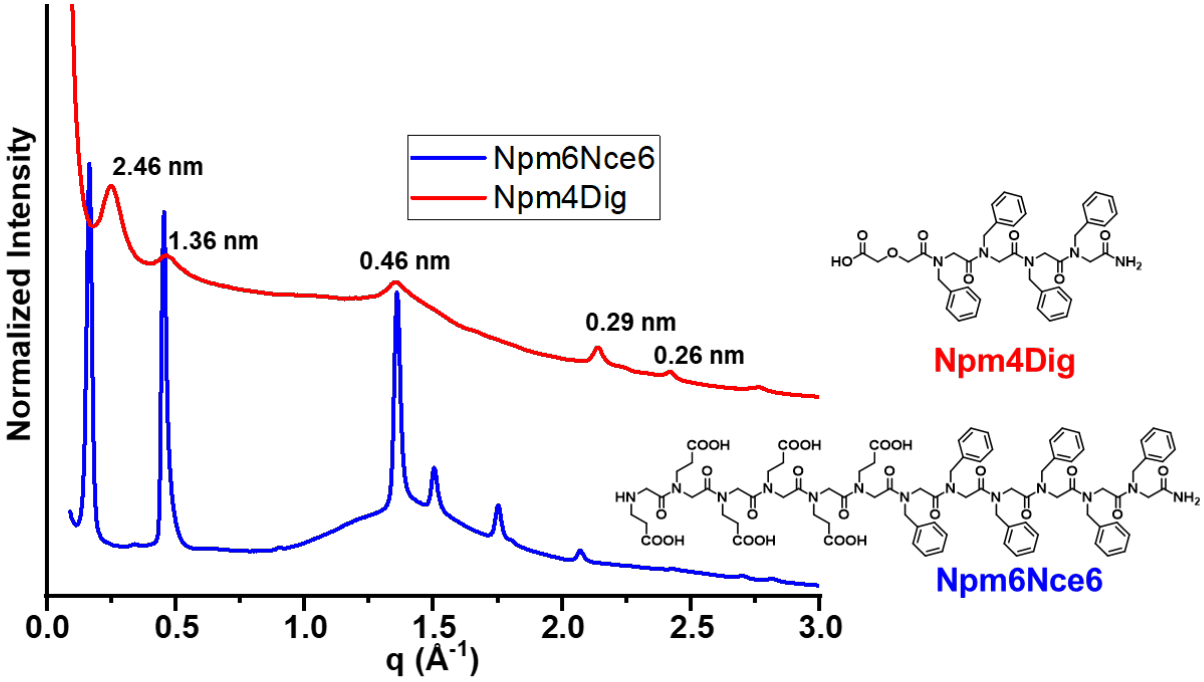

Supplementary Figure 11 | XRD data of nanohelices assembled from Npm4Dig and nanosheets assembled from Npm6Nce6.

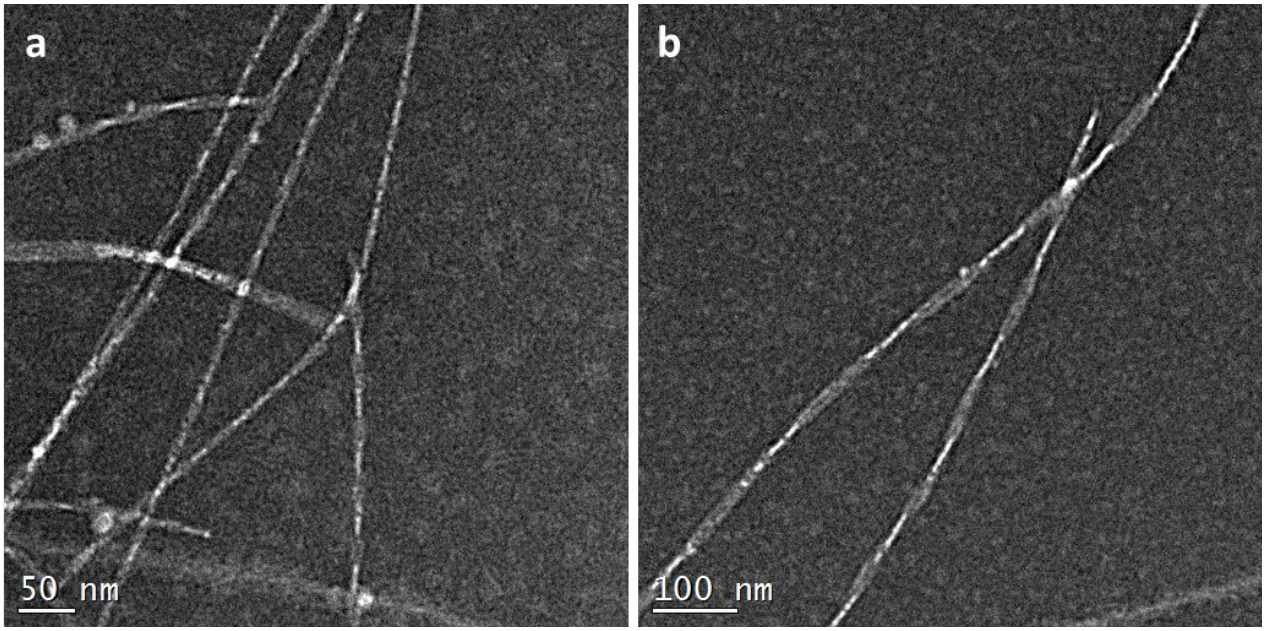

Supplementary Figure 12 | Pepotid nanohelices self-assembled from peptoid Npm4Dig with different ratios of CH_3CN and H_2O. a) Negatively stained TEM image of nanohelices self-assembled from Npm4Dig in 1:2 CH_3CN&H_2O, (b) Negatively stained TEM image of nanohelices self-assembled from Npm4Dig in 2:1 CH_3CN&H_2O.

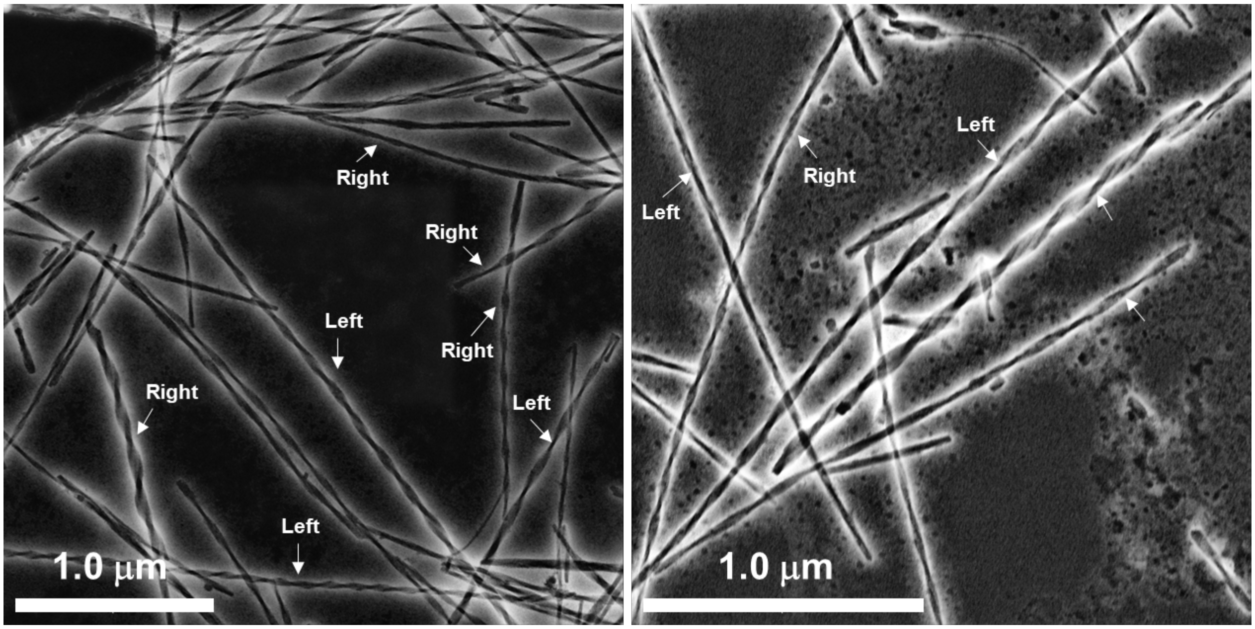

Supplementary Figure 13 | . Negative-stained ADF-STEM images of Npm4Dig nanohelices self-assembled at pH = 4. The ratio of left- and right-hand twists is close to 1:1 in both cases.

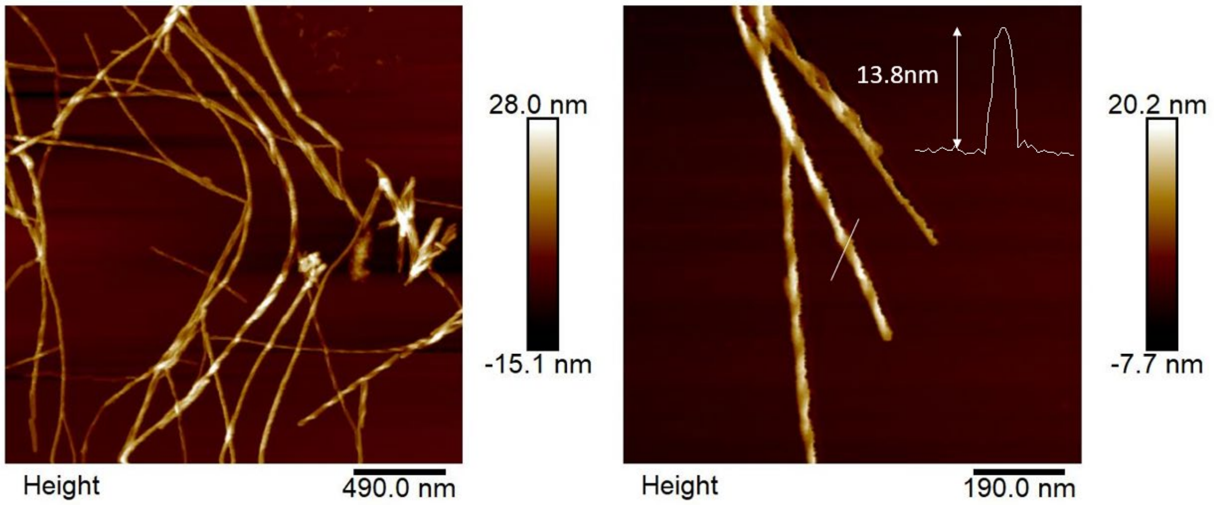

Supplementary Figure 14 | . AFM images of Npm4Dig nanohelices self-assembled at pH = 4. Height distribution shown in Figure 3e is calculated from these nanohelices. Inset: height profile along the white line in the right image showing the height of peptoid nanohelix.

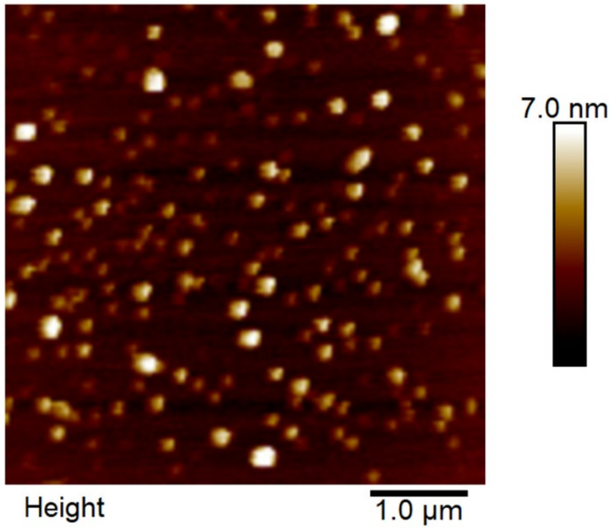

Supplementary Figure 15 | . AFM image showing amorphous aggregates formed by Npm4Dig at pH = 12.

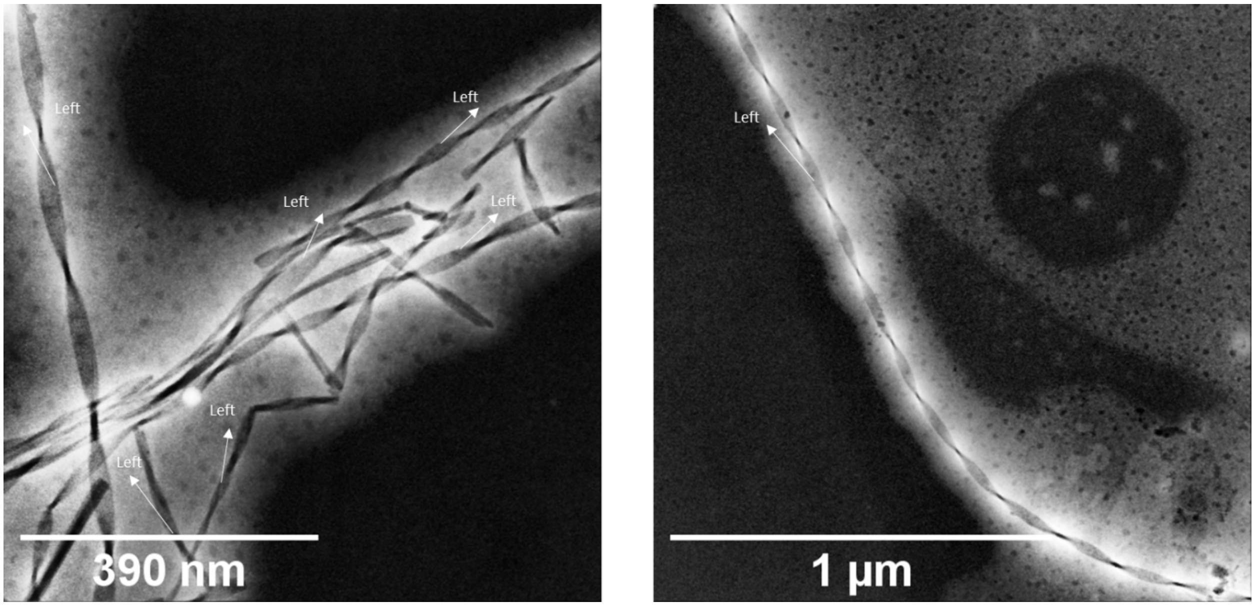

Supplementary Figure 16 | . Negatively-stained ADF-STEM images of left-handed Npm4-D-Ala nanohelices self-assembled at pH = 4.

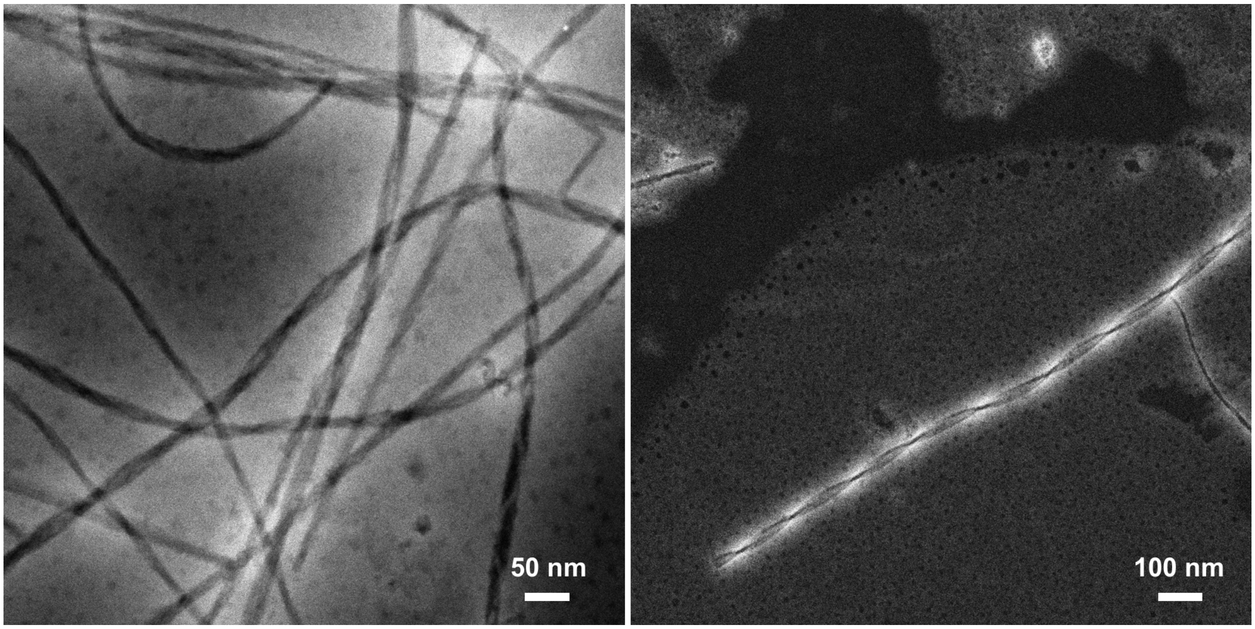

Supplementary Figure 17 | . Negatively-stained ADF-STEM images of right-handed Npm4-L-Ala nanohelices self-assembled at pH = 4.

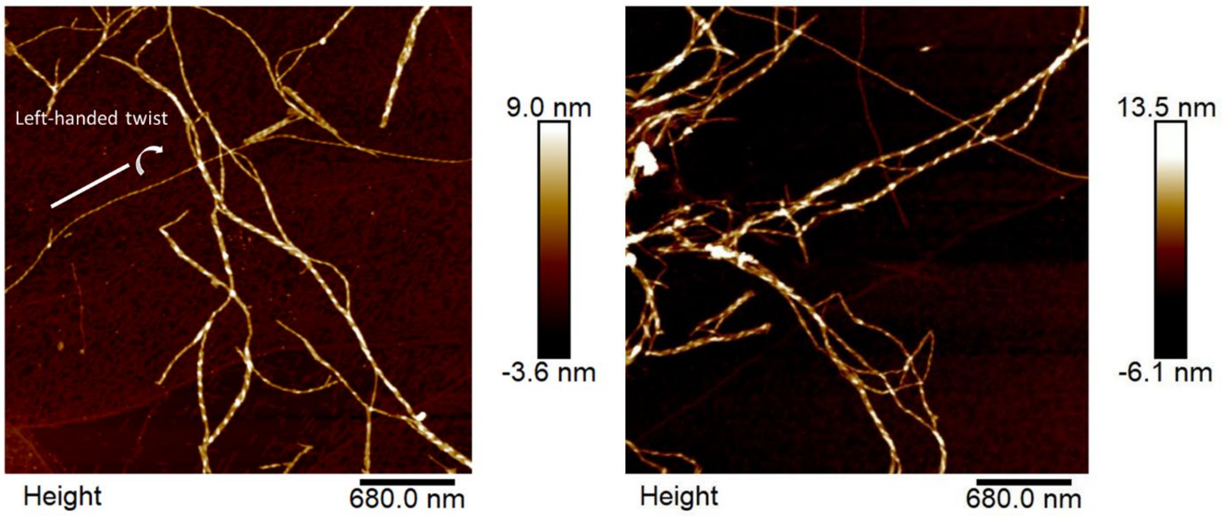

Supplementary Figure 18 | . AFM images of the left-handed nanohelices self-assembled from Npm4-D-Ala at pH = 4 Large scale images demonstrate all left-handed twists.

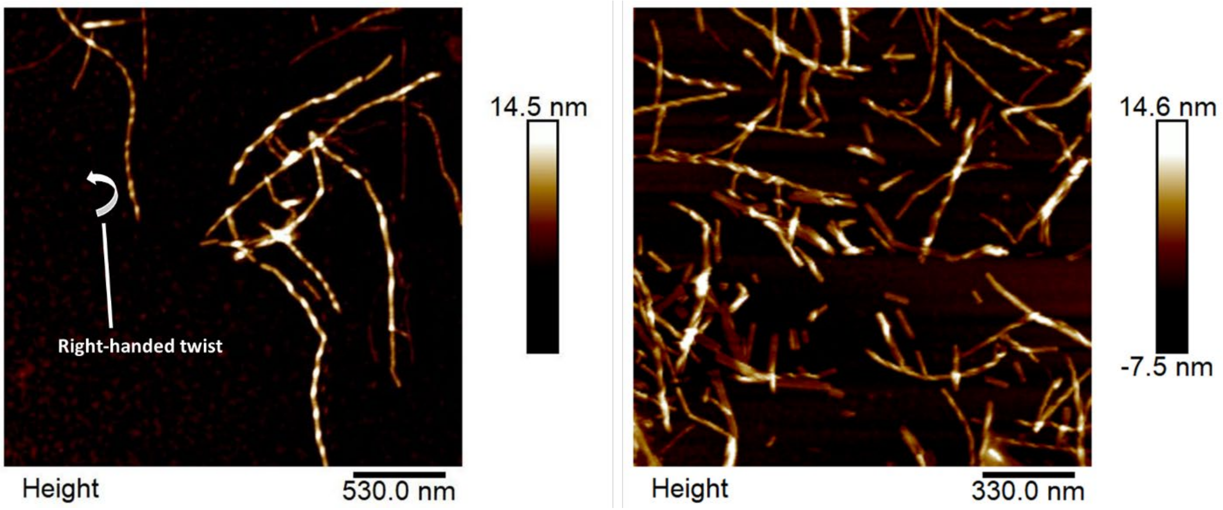

Supplementary Figure 19 | . AFM images of the right-handed nanohelices self-assembled from Npm4-L-Ala at pH = 4. Large scale-images demonstrate all right-handed twists.

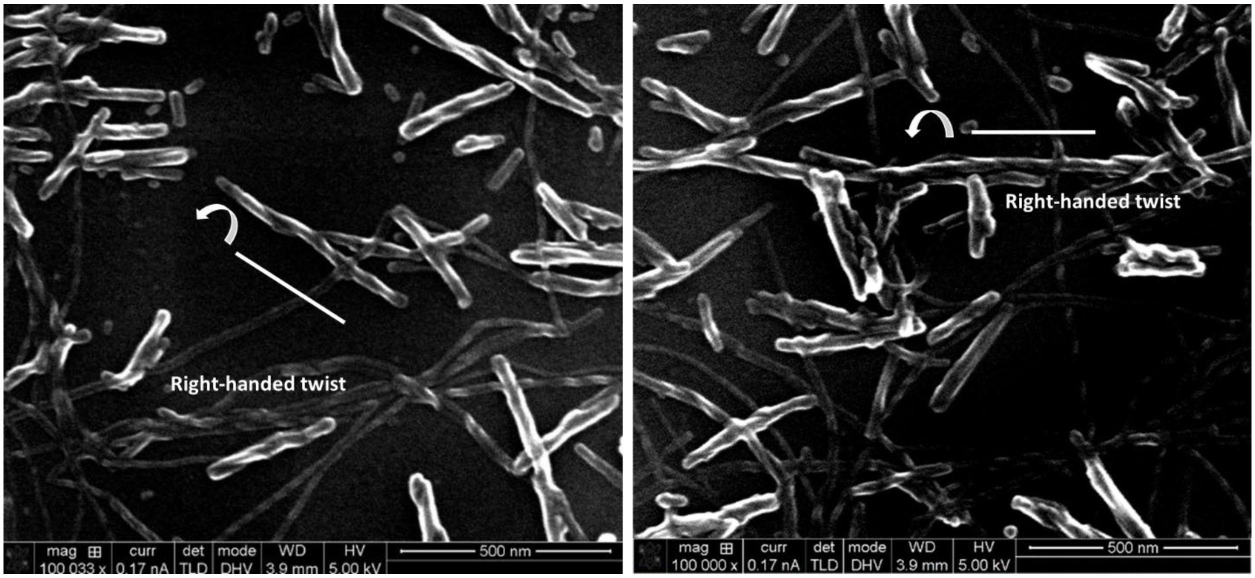

Supplementary Figure 20 | SEM images of the right-handed nanohelices self-assembled from Npm4-L-Ala at pH = 4.

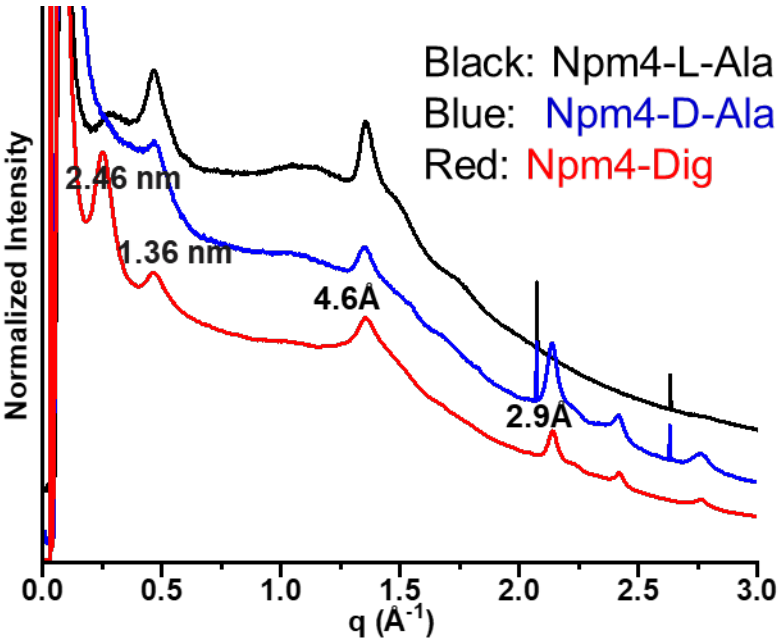

Supplementary Figure 21 | XRD data of peptoid nanohelices assembled from Npm4-L-Ala, from Npm4-D-Ala, and from Npm4Dig. Similar peaks at *d* = 1.36 nm and 4.6 Å indicate that these peptoid helices exhibit a similar framework structure.

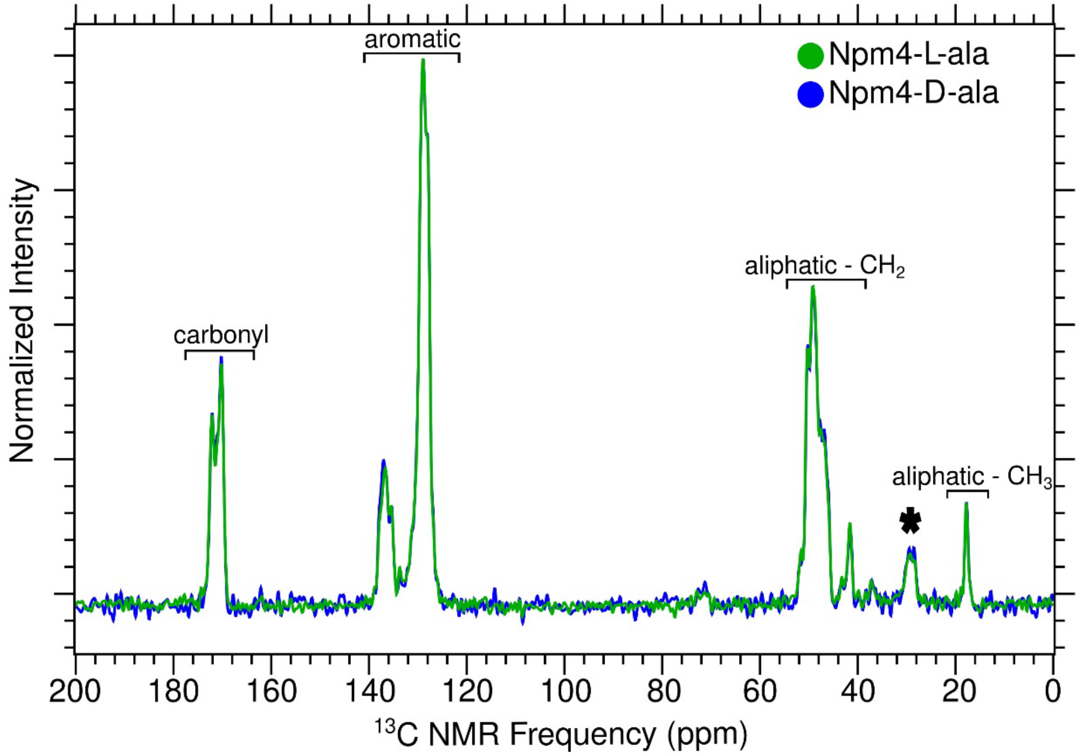

Supplementary Figure 22 | Solid-state C^{13} NMR data of nanohelices assembled from Npm4-L-Ala and Npm4-D-Ala. The stars indicate positions of magic-angle spinning sidebands.

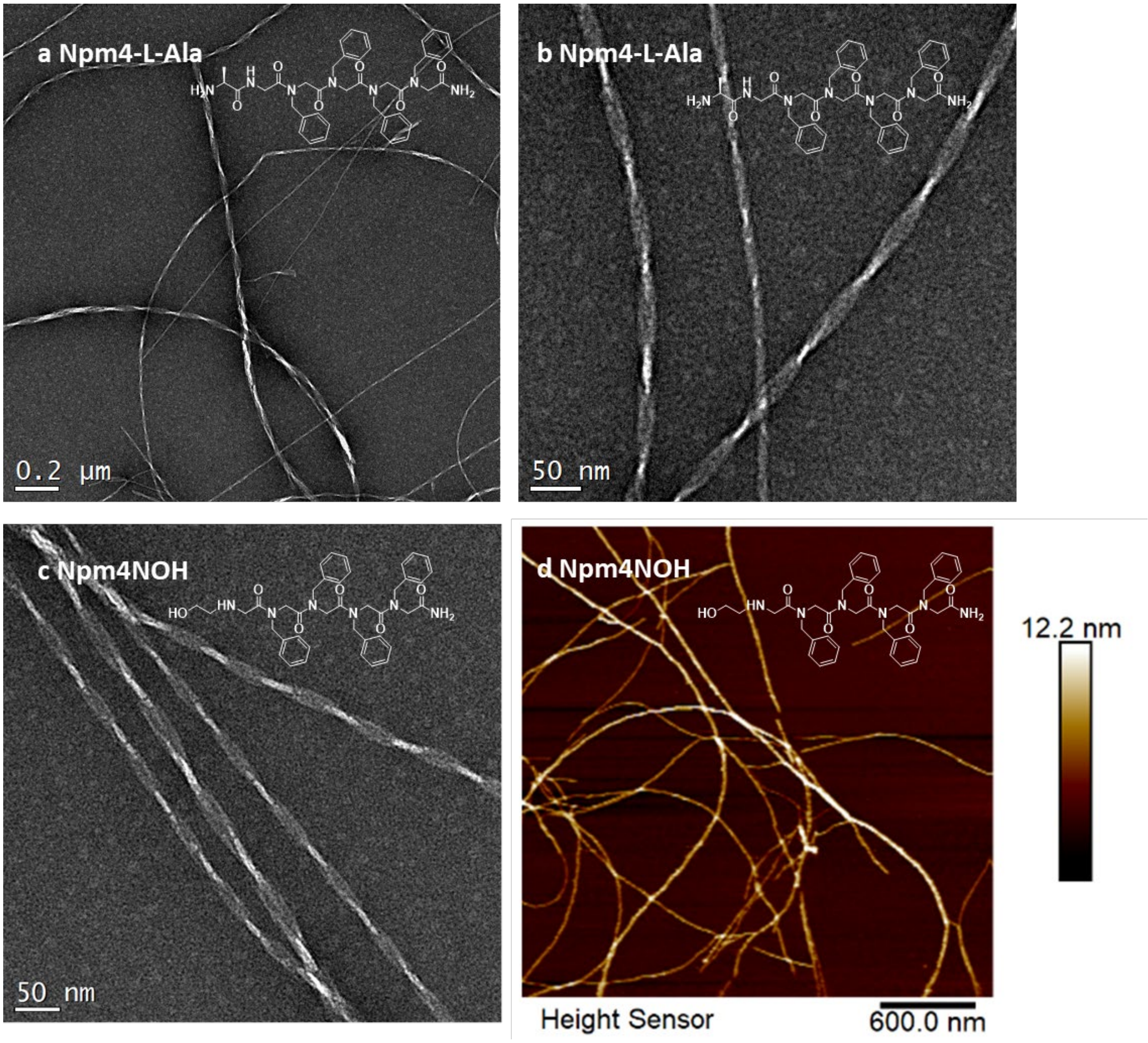

Supplementary Figure 23 | Pepotid nanohelices self-assembled from peptoids with different polar domains. a,b) Negatively stained TEM images of Npm4-L-Ala nanohelices self-assembled at pH = 10. **c**) Negatively stained TEM image of Npm4Noh nanohelices self-assembled at pH = 4. **d**) AFM image of Npm4Noh nanohelices self-assembled at pH = 4. The insert in each figure is the chemical structure of the corresponding peptoid.

Supplementary References.

1 Jin, H. *et al.* Designable and dynamic single-walled stiff nanotubes assembled from sequence-defined peptoids. *Nat. Commun.* **9**, 270 (2018). https://doi.org:10.1038/s41467-017-02059-1

2 Jin, H. *et al.* Highly stable and self-repairing membrane-mimetic 2D nanomaterials assembled from lipid-like peptoids. *Nat. Commun.* **7**, 12252 (2016). https://doi.org:10.1038/ncomms12252

3 Hanwell, M. D. *et al.* Avogadro: an advanced semantic chemical editor, visualization, and analysis platform. *Journal of Cheminformatics* **4**, 17 (2012). https://doi.org:10.1186/1758-2946-4-17

4 Weiser, L. J. & Santiso, E. E. A CGenFF-based force field for simulations of peptoids with both cis and trans peptide bonds. *J. Comput. Chem.* **40**, 1946-1956 (2019). https://doi.org:https://doi.org/10.1002/jcc.25850

5 Mackerell Jr, A. D., Feig, M. & Brooks Iii, C. L. Extending the treatment of backbone energetics in protein force fields: Limitations of gas-phase quantum mechanics in reproducing protein conformational distributions in molecular dynamics simulations. *J. Comput. Chem.* **25**, 1400-1415 (2004). https://doi.org:https://doi.org/10.1002/jcc.20065

6 Best, R. B. *et al.* Optimization of the Additive CHARMM All-Atom Protein Force Field Targeting Improved Sampling of the Backbone φ, ψ and Side-Chain χ1 and χ2 Dihedral Angles. *J. Chem. Theory Comput.* **8**, 3257-3273 (2012). https://doi.org:10.1021/ct300400x

7 Williams, S. J. Methoxyacetic acid esters: Applications in protecting group and glycosylation chemistry. *Carbohydr. Res.* **486**, 107848 (2019). https://doi.org:https://doi.org/10.1016/j.carres.2019.107848

8 Jorgensen, W. L., Chandrasekhar, J., Madura, J. D., Impey, R. W. & Klein, M. L. Comparison of simple potential functions for simulating liquid water. *J. Chem. Phys.* **79**, 926-935 (1983). https://doi.org:10.1063/1.445869

9 Martínez, L., Andrade, R., Birgin, E. G. & Martínez, J. M. PACKMOL: A package for building initial configurations for molecular dynamics simulations. *J. Comput. Chem.* **30**, 2157-2164 (2009). https://doi.org:https://doi.org/10.1002/jcc.21224

10 Abraham, M. J. *et al.* GROMACS: High performance molecular simulations through multi-level parallelism from laptops to supercomputers. *SoftwareX* **1-2**, 19-25 (2015). https://doi.org:https://doi.org/10.1016/j.softx.2015.06.001

11 Humphrey, W., Dalke, A. & Schulten, K. VMD: Visual molecular dynamics. *Journal of Molecular Graphics* **14**, 33-38 (1996). https://doi.org:https://doi.org/10.1016/0263-7855(96)00018-5

12 Zhao, M. *et al.* Hierarchical Self-Assembly Pathways of Peptoid Helices and Sheets. *Biomacromolecules* **23**, 992-1008 (2022). https://doi.org:10.1021/acs.biomac.1c01385

13 Bussi, G., Donadio, D. & Parrinello, M. Canonical sampling through velocity rescaling. *J. Chem. Phys.* **126**, 014101 (2007). https://doi.org:10.1063/1.2408420

14 Berendsen, H. J. C., Postma, J. P. M., van Gunsteren, W. F., DiNola, A. & Haak, J. R. Molecular dynamics with coupling to an external bath. *J. Chem. Phys.* **81**, 3684-3690 (1984). https://doi.org:10.1063/1.448118

15 Nosé, S. A unified formulation of the constant temperature molecular dynamics methods. *J. Chem. Phys.* **81**, 511-519 (1984). https://doi.org:10.1063/1.447334

16 Parrinello, M. & Rahman, A. Polymorphic transitions in single crystals: A new molecular dynamics method. *J. Appl. Phys.* **52**, 7182-7190 (1981). https://doi.org:10.1063/1.328693

17 scikit-spatial: Spatial objects and computations based on NumPy arrays.

18 Smith, J. S. *et al.* A phylogenetically conserved NAD+-dependent protein deacetylase activity in the Sir2 protein family. *PNAS* **97**, 6658-6663 (2000). https://doi.org:10.1073/pnas.97.12.6658

19 Nyrkova, I. A., Semenov, A. N., Aggeli, A. & Boden, N. Fibril stability in solutions of twisted -sheet peptides: a new kind of micellization in chiral systems. *Eur. Phys. J. B.* **17**, 481-497 (2000). https://doi.org:10.1007/s100510070127

20 Rüter, A., Kuczera, S., Pochan, D. J. & Olsson, U. Twisted Ribbon Aggregates in a Model Peptide System. *Langmuir* **35**, 5802-5808 (2019). https://doi.org:10.1021/acs.langmuir.8b03886

21 Rolo, L. I., Caço, A. I., Queimada, A. J., Marrucho, I. M. & Coutinho, J. A. P. Surface Tension of Heptane, Decane, Hexadecane, Eicosane, and Some of Their Binary Mixtures. *Journal of Chemical & Engineering Data* **47**, 1442-1445 (2002). https://doi.org:10.1021/je025536+
22 Cai, W. *et al.* Force-Induced Transition of π–π Stacking in a Single Polystyrene Chain. *J. Am. Chem. Soc.* **141**, 9500-9503 (2019). https://doi.org:10.1021/jacs.9b03490
