# Supplementary material for: Assembly of short amphiphilic peptoids into nanohelices with controllable supramolecular chirality
Source: Nat Commun. 2024 Apr 16;15:3264. doi: 10.1038/s41467-024-46839-y (PMC11021492; doi:10.1038/s41467-024-46839-y)
Supplement: Supplementary file 3 — Description of Additional Supplementary Files [file 41467_2024_46839_MOESM3_ESM.pdf]

Description of Additional Supplementary Files

File Name: Supplementary Data 1
Description: The coordinate file (GRO) for the initial configuration of the whole simulation box.

File Name: Supplementary Data 2
Description: The coordinate file (GRO) for the final configuration (20 ns) of the whole simulation box.
